# Complete mitochondrial genome of *Scincella modesta* (Squamata: Scincidae) and phylogenetic analysis

**DOI:** 10.1080/23802359.2019.1703579

**Published:** 2020-01-07

**Authors:** Jun Zhong, Li Ma, Kun Guo, Yu Du

**Affiliations:** a College of Life Sciences, Jiangsu Key Laboratory for Biodiversity and Biotechnology, Nanjing Normal University, Nanjing, P. R. China;; b Faculty of Ecology, Lishui University, Lishui, P. R. China;; c Hainan Key Laboratory for Herpetological Research, College of Fisheries and Life Science, Hainan Tropical Ocean University, Sanya, P. R. China

**Keywords:** Reptilia, *Scincella modesta*, mitogenome, phylogenetic analysis

## Abstract

We sequenced and annotated the complete mitochondrial genome (mitogenome) of *Scincella modesta* (Squamata: Scincidae). This mitogenome was 17,466 bp long and encoded 13 protein-coding genes (PCGs), 22 transfer RNA genes, 2 ribosomal RNA genes, and 2 non-coding regions. The overall nucleotide composition was 31.8% of A, 14.5% of G, 27.2% of T, and 26.5% of C. Phylogenetic analysis using Bayesian Inference (BI) validated the taxonomic status of *S. modesta*, exhibiting the close relationship with the other two species from the genus *Scincella*.

*Scincella modesta* (Squamata: Scincidae) is a small lizard, which is mainly distributed in the south of the Yangtze River of China. In this study, we sequenced the mitochondrial genome of *S. modesta* (GenBank accession No. MN702771), representing the third mitochondrial genome from the genus *Scincella*.

The specimens of *S. modesta* were collected in Nanjing, China. The collected specimens were stored in 95% ethanol at temperature −20 °C. The specimen and its DNA were stored in the Research center of herpetology, Nanjing Normal University, Nanjing, the Accession number of the specimen was NB2017030715. Whole genomic DNA was extracted from tail tissues of each specimen using a Wizard^®^ Genomic DNA Purification Kit (Promega, Madison, WI, USA) according to the manufacturer’s instructions. The genomic DNA was sequenced using the Hiseq2000 platform (Illumina Inc., San Diego, CA, USA). The mitogenome of *Scincella huanrenensis* (GenBank accession No. KU507306) was employed as the reference sequence (Park et al. 2016). Mitochondrial genome was assembled by Geneious version 9.0.4 (Biomatters Ltd., Auckland, New Zealand) (https://www.geneious.com), and annotated using MITOS Web Server (Bernt et al. 2013).

We obtained complete mitogenome of *S. modesta* with 17,466 bp long. This mitogenome encoded 13 protein-coding genes (PCGs), 22 tRNAs, 2 rRNAs (*rrnL* and *rrnS*), and 2 non-coding regions of an L-strand replication origin and a displacement loop region. The overall nucleotide composition was 31.8% of A, 14.5% of G, 27.2% of T, and 26.5% of C. The gene arrangement pattern and transcription directions were identical to previous studies in Lacertilia (Kim et al. 2016; Song et al. 2016). Among the 13 PCGs, the *ATP8* was the shortest, while the *ND5* was the longest. All PCGs initiated with ATG as a start codon, except for *COI* gene, which began with GTG. Eight genes (*ND1*, *COI*, *ATP8*, *ATP6*, *ND4L*, *ND5*, *ND6*, and *Cytb*) end with complete stop codons (TAA, AGA, and AGG), and the other five genes end with T as the incomplete stop codons, which were presumably completed as TAA by post-transcriptional polyadenylation (Anderson et al. 1981).

To validate the phylogenetic position of *S. modesta*, the Bayesian Inference (BI) tree was constructed on CIPRES Portal using 13 mitochondrial PCGs from mitogenomes of 12 species. We used the best-fit partitioning scheme and partition-specific models recommended by PartitionFinder (Lanfear et al. 2012). As shown in Figure 1, *S. modesta* was positioned near *S. vandenburghi* within the genus *Scincella*. The phylogenetic analysis result was consistent with the previous research. It indicated that our new determined mitogenome sequences could meet the demands and explain some evolution issues.

**Figure 1.**
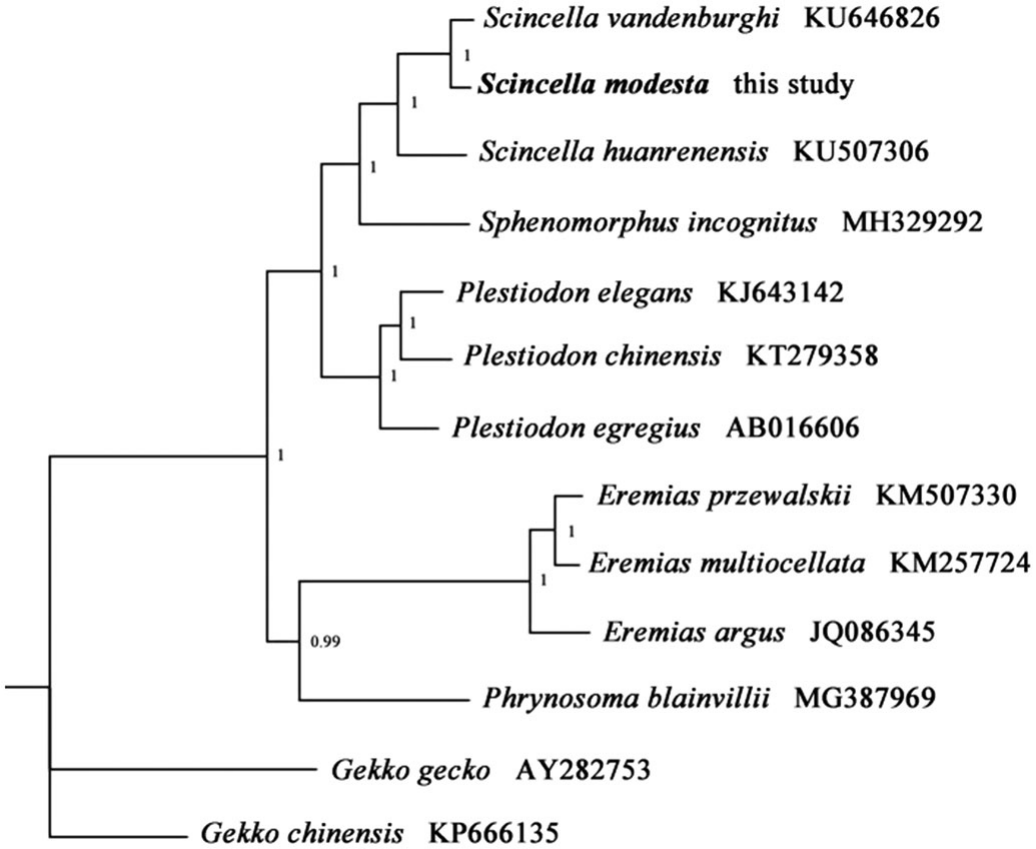
Phylogenetic tree obtained from BI analysis based on 13 concatenated mitochondrial PCGs. Numbers on node are posterior probability (PP).
